# Supplementary material for: Phosphatidylserine-exposing extracellular vesicles in body fluids are an innate defence against apoptotic mimicry viral pathogens
Source: Nat Microbiol. 2024 Mar 25;9(4):905–21. doi: 10.1038/s41564-024-01637-6 (PMC10994849; doi:10.1038/s41564-024-01637-6)
Supplement: Supplementary file 1 — Supplementary Tables 1 and 2, References, Gating strategy, Uncut western blots and List of reagents. [file 41564_2024_1637_MOESM1_ESM.pdf]

nature microbiology

Article https://doi.org/10.1038/s41564-024-01637-6

Phosphatidylserine-exposing extracellular vesicles in body fluids are an innate defence against apoptotic mimicry viral pathogens

In the format provided by the authors and unedited

Supplementary Table 1: Shedding into body fluids and modes of human to human transmission for viruses using or not using viral apoptotic mimicry. VAM – using viral apoptotic mimicry as a primary entry mechanism.

Virus	VAM	Sexual transmission		Oral transmission		Blood-borne	
		Semen	Frequency	Saliva	Frequency	Blood	Frequency
Zika	Yes[1]	Yes[2–4]	Rare[2,4]	Yes[4]	None reported	Yes[5]	Dominant via mosquito vectors[6]
Dengue	Yes[1,10]	Yes[11]	Anecdotally[11]	Yes[12,13]	None reported	Yes[14]	Dominant via mosquito vectors[6,15]
West Nile	Yes[1,10]	None reported	None reported	Yes[13]	None reported	Low[16]	Dead end hosts, transfusion possible[16,17]
Chikungunya	Yes[1,10]	Yes[3]	None reported[3]	Yes[13,19]	None reported	Yes[20]	Dominant via mosquito vectors[15,21]
Ebola	Yes[1,10]	Yes[3]	Rare[23]	Yes[13,23,24]	Possible route [23]	Yes[23,24]	Possible route[23]
SARS-CoV-2	No[1,25]	Anecdotally [26]	None reported	Yes[27]	Common[28,29]	Low[30]	No evidence[31]
HIV-1	No[1]	Yes[3]	Dominant route[34]	Yes[35]	Unlikely[36]	Yes[36]	Possible route[34,36]
CMV	No[39]	Yes[3]	Possible route[40]	Yes[13,41]	Common route[41]	Yes[41]	Possible route[41,42]
HSV-1	No[10,43]	Yes[3]	Possible route[44]	Yes[45 13]	Dominant route[45]	Low[42]	No evidence[42]
HSV-2	No[43]	Yes[3]	Dominant route[44]	Yes[13]	Possible route[44]	Low[42]	No evidence[42]
Hepatitis C	No[46]	Yes[3]	Possible route[47–49]	Yes[13,50]	None reported[50]	Yes[51]	Possible route[51]

Virus	VAM	Breast feeding		Urine	
		Milk	Frequency	Urine	Frequency
Zika	Yes[1]	Yes[7]	Rare[4,8,9]	Yes[5]	None reported
Dengue	Yes[1,10]	Yes[7]	None reported[7]	Yes[11]	None reported
West Nile	Yes[1,10]	Yes[7]	None reported[7]	Yes[18]	None reported
Chikungunya	Yes[1,10]	Anecdotally [22]	None reported	Yes[19]	None reported
Ebola	Yes[1,10]	Yes[23,24]	Rare[23]	Yes[23,24]	Possible route[23]
SARS-CoV-2	No[1,25]	Yes[32]	None reported [32]	Yes[26,33]	None reported
HIV-1	No[1]	Yes[37]	Possible route[37,38]	Yes[36]	Unlikely[36]
CMV	No[39]	Yes[41]	Possible route[38,41]	Yes[41]	Possible route[41]
HSV-1	No[10,43]	n/a	None reported	None reported	None reported
HSV-2	No[43]	n/a	None reported	None reported	None reported
Hepatitis C	No[46]	Yes[52]	Rare[52]	Yes[53]	None reported

Supplementary Table 2: Purified EVs from body fluids and cell culture supernatant, related to Figure 3. The number of preparations refers to unique isolation procedures performed per source; for sources with multiple preparations the range of donors, volume, particle concentration, concentration factor and particle recovery are given. Recovery of particles is calculated by NTA-tracked particles in the source fluid (after centrifugation) and the final EV preparation; due to presence of non-EV-particles especially in the source fluid which may be tracked by NTA, this does not necessarily reflect the recovery of EVs specifically. N/A = not applicable. * semen used for preparation of seminal plasma was obtained as excess material from a fertility clinic as unlabeled aliquots; the number of donors shown assumes that all aliquots are from separate donors, but repeated donations and multiple aliquots per donation are possible and cannot be verified.

Source	Isolations	Donors	Source volume [ml]	Particles/ml source	Particles/ml EV preparation	Conc. by volume	Recovery particles [%]
**Semen**	3	35 - 100*	33 - 90	$3.7 - 4.0 \times 10^{12}$	$4.3 - 9.9 \times 10^{12}$	8 - 11x	10.3 - 25.2
**Saliva**	5	9 - 10	100 - 300	$3.3 - 8.5 \times 10^{10}$	$1.7 - 9.9 \times 10^{11}$	100 - 429x	1.6 - 18.2
**Urine**	1	6	3,125	8.2×10^{9}	4.4×10^{12}	2,232x	23.9
**Breast milk**	1	1	49	2.3×10^{12}	2.5×10^{13}	33x	33.2
**Blood**	3	N/A	20 - 40	2.2×10^{12}	1.3×10^{12}	20x	3.0

References

1. Bohan, D. & Maury, W. Enveloped RNA virus utilization of phosphatidylserine receptors: Advantages of exploiting a conserved, widely available mechanism of entry. *PLOS Pathog.* **17**, e1009899 (2021).
2. Counotte, M. J. *et al.* Sexual transmission of Zika virus and other flaviviruses: A living systematic review. *PLOS Med.* **15**, e1002611 (2018).
3. Salam, A. P. & Horby, P. W. The Breadth of Viruses in Human Semen. *Emerg. Infect. Dis.* **23**, 1922–1924 (2017).
4. Grischott, F., Puhan, M., Hatz, C. & Schlagenhauf, P. Non-vector-borne transmission of Zika virus: A systematic review. *Travel Med. Infect. Dis.* **14**, 313–330 (2016).
5. Paz-Bailey, G. *et al.* Persistence of Zika Virus in Body Fluids — Final Report. *N. Engl. J. Med.* **379**, 1234–1243 (2018).
6. Pierson, T. C. & Diamond, M. S. The continued threat of emerging flaviviruses. *Nat. Microbiol.* **5**, 796–812 (2020).
7. Mann, T. Z. *et al.* Breast milk transmission of flaviviruses in the context of Zika virus: A systematic review. *Paediatr. Perinat. Epidemiol.* **32**, 358–368 (2018).
8. Blohm, G. M. *et al.* Evidence for Mother-to-Child Transmission of Zika Virus Through Breast Milk. *Clin. Infect. Dis.* **66**, 1120–1121 (2018).
9. Cavalcanti, M. G. *et al.* Zika virus shedding in human milk during lactation: an unlikely source of infection? *Int. J. Infect. Dis.* **57**, 70–72 (2017).
10. Amara, A. & Mercer, J. Viral apoptotic mimicry. *Nat. Rev. Microbiol.* **13**, 461–469 (2015).
11. Grobusch, M. P. *et al.* Can dengue virus be sexually transmitted? *Travel Med. Infect. Dis.* **38**, 101753 (2020).
12. Humaidi, M., Tien, W. P., Yap, G., Chua, C. R. & Ng, L. C. Non-Invasive Dengue Diagnostics—The Use of Saliva and Urine for Different Stages of the Illness. *Diagnostics* **11**, 1345 (2021).
13. Limeres Posse, J., Diz Dios, P. & Scully, C. Viral Diseases Transmissible by Kissing. in *Saliva Protection and Transmissible Diseases* 53–92 (Elsevier, 2017). doi:10.1016/B978-0-12-813681-2.00004-4.
14. Matangkasombut, P. *et al.* Dengue viremia kinetics in asymptomatic and symptomatic infection. *Int. J. Infect. Dis.* **101**, 90–97 (2020).
15. Kuno, G. & Chang, G.-J. J. Biological Transmission of Arboviruses: Reexamination of and New Insights into Components, Mechanisms, and Unique Traits as Well as Their Evolutionary Trends. *Clin. Microbiol. Rev.* **18**, 608–637 (2005).
16. Colpitts, T. M., Conway, M. J., Montgomery, R. R. & Fikrig, E. West Nile Virus: Biology, Transmission, and Human Infection. *Clin. Microbiol. Rev.* **25**, 635–648 (2012).
17. Petersen, L. R., Brault, A. C. & Nasci, R. S. West Nile Virus: Review of the Literature. *JAMA* **310**, 308 (2013).
18. Barzon, L. *et al.* Isolation of West Nile Virus from Urine Samples of Patients with

Acute Infection. *J. Clin. Microbiol.* **52**, 3411–3413 (2014).

19. Martins, E. B. *et al.* Detection of Chikungunya virus in bodily fluids: The INOVACHIK cohort study. *PLoS Negl. Trop. Dis.* **16**, e0010242 (2022).
20. Burt, F. J. *et al.* Chikungunya virus: an update on the biology and pathogenesis of this emerging pathogen. *Lancet Infect. Dis.* **17**, e107–e117 (2017).
21. Bala Murugan, S. & Sathishkumar, R. Chikungunya infection: A potential re-emerging global threat. *Asian Pac. J. Trop. Med.* **9**, 933–937 (2016).
22. Campos, G. S. *et al.* First Detection of Chikungunya Virus in Breast Milk. *Pediatr. Infect. Dis. J.* **36**, 1015–1017 (2017).
23. Jacob, S. T. *et al.* Ebola virus disease. *Nat. Rev. Dis. Prim.* **6**, 13 (2020).
24. Vetter, P. *et al.* Ebola Virus Shedding and Transmission: Review of Current Evidence. *J. Infect. Dis.* **214**, S177–S184 (2016).
25. Hoffmann, M. *et al.* SARS-CoV-2 Cell Entry Depends on ACE2 and TMPRSS2 and Is Blocked by a Clinically Proven Protease Inhibitor. *Cell* **181**, 271-280.e8 (2020).
26. Gonzalez, D. C. *et al.* A Systematic Review on the Investigation of SARS-CoV-2 in Semen. *Res. Reports Urol.* **Volume 12**, 615–621 (2020).
27. Huang, N. *et al.* SARS-CoV-2 infection of the oral cavity and saliva. *Nat. Med.* **27**, 892–903 (2021).
28. Greenhalgh, T. *et al.* Ten scientific reasons in support of airborne transmission of SARS-CoV-2. *Lancet* **397**, 1603–1605 (2021).
29. Jayaweera, M., Perera, H., Gunawardana, B. & Manatunge, J. Transmission of COVID-19 virus by droplets and aerosols: A critical review on the unresolved dichotomy. *Environ. Res.* **188**, 109819 (2020).
30. Li, Y. & Li, J. Z. SARS-CoV-2 Virology. *Infect. Dis. Clin. North Am.* **36**, 251–265 (2022).
31. Mawalla, W. F., Njiro, B. J., Bwire, G. M., Nasser, A. & Sunguya, B. No evidence of SARS-CoV-2 transmission through transfusion of human blood products: A systematic review. *eJHaem* **2**, 601–606 (2021).
32. Centeno-Tablante, E. *et al.* Transmission of SARS-CoV-2 through breast milk and breastfeeding: a living systematic review. *Ann. N. Y. Acad. Sci.* **1484**, 32–54 (2021).
33. Jones, D. L. *et al.* Shedding of SARS-CoV-2 in feces and urine and its potential role in person-to-person transmission and the environment-based spread of COVID-19. *Sci. Total Environ.* **749**, 141364 (2020).
34. Deeks, S. G., Overbaugh, J., Phillips, A. & Buchbinder, S. HIV infection. *Nat. Rev. Dis. Prim.* **1**, 15035 (2015).
35. Liuzzi, G. *et al.* Analysis of HIV-1 load in blood, semen and saliva: evidence for different viral compartments in a cross-sectional and longitudinal study. *AIDS* **10**, F51–F56 (1996).
36. Levy, J. A. Pathogenesis of human immunodeficiency virus infection. *Microbiol. Rev.* **57**, 183–289 (1993).
37. John-Stewart, G. *et al.* Breast-feeding and Transmission of HIV-1. *JAIDS J. Acquir. Immune Defic. Syndr.* **35**, 196–202 (2004).

38. Hamprecht, K. *et al.* Epidemiology of transmission of cytomegalovirus from mother to preterm infant by breastfeeding. *Lancet* **357**, 513–518 (2001).
39. Soares, M. M., King, S. W. & Thorpe, P. E. Targeting inside-out phosphatidylserine as a therapeutic strategy for viral diseases. *Nat. Med.* **14**, 1357–1362 (2008).
40. Handsfield, H. H. *et al.* Cytomegalovirus Infection in Sex Partners: Evidence for Sexual Transmission. *J. Infect. Dis.* **151**, 344–348 (1985).
41. Plosa, E. J., Esbenshade, J. C., Fuller, M. P. & Weitkamp, J.-H. Cytomegalovirus Infection. *Pediatr. Rev.* **33**, 156–163 (2012).
42. Juhl, D. *et al.* Detection of herpes simplex virus DNA in plasma of patients with primary but not with recurrent infection: implications for transfusion medicine? *Transfus. Med.* **20**, 38–47 (2010).
43. Madavaraju, K., Koganti, R., Volety, I., Yadavalli, T. & Shukla, D. Herpes Simplex Virus Cell Entry Mechanisms: An Update. *Front. Cell. Infect. Microbiol.* **10**, 1–18 (2021).
44. Brugha, R., Keersmaekers, K., Renton, A. & Meheus, A. Genital herpes infection: a review. *Int. J. Epidemiol.* **26**, 698–709 (1997).
45. Atyeo, N., Rodriguez, M. D., Papp, B. & Toth, Z. Clinical Manifestations and Epigenetic Regulation of Oral Herpesvirus Infections. *Viruses* **13**, 681 (2021).
46. Barth, H., Liang, T. J. & Baumert, T. F. Hepatitis C virus entry: Molecular biology and clinical implications. *Hepatology* **44**, 527–535 (2006).
47. Rooney, G. & Gilson, R. J. Sexual transmission of hepatitis C virus infection. *Sex. Transm. Infect.* **74**, 399–404 (1998).
48. Terrault, N. A. Sexual activity as a risk factor for hepatitis C. *Hepatology* **36**, s99–s105 (2002).
49. Edwards, S. & Carne, C. Oral sex and the transmission of viral STIs. *Sex. Transm. Infect.* **74**, 6–10 (1998).
50. Ferreiro, M., Dios, P. & Scully, C. Transmission of hepatitis C virus by saliva? *Oral Dis.* **11**, 230–235 (2005).
51. Prati, D. Transmission of hepatitis C virus by blood transfusions and other medical procedures: A global review. *J. Hepatol.* **45**, 607–616 (2006).
52. Bhola, K. & McGuire, W. Does avoidance of breast feeding reduce mother-to-infant transmission of hepatitis C virus infection? *Arch. Dis. Child.* **92**, 365–366 (2007).
53. Lu, T. *et al.* Quantitative detection of hepatitis C virus RNA in urine of patients with chronic hepatitis C using a novel real-time PCR assay. *J. Med. Virol.* **91**, 115–123 (2019).
54. Choi, J.-Y. *et al.* A novel fluorescence assay for measuring phosphatidylserine decarboxylase catalysis. *J. Biol. Chem.* **293**, 1493–1503 (2018).
55. Choi, J.-Y., Augagneur, Y., Mamoun, C. Ben & Voelker, D. R. Identification of Gene Encoding Plasmodium knowlesi Phosphatidylserine Decarboxylase by Genetic Complementation in Yeast and Characterization of in Vitro Maturation of Encoded Enzyme. *J. Biol. Chem.* **287**, 222–232 (2012).
56. Proffitt, M. R. & Schindler, S. A. Rapid detection of HSV with an enzyme-linked

virus inducible system™ (ELVIS™) employing a genetically modified cell line. *Clin. Diagn. Virol.* **4**, 175–182 (1995).
57. Tsetsarkin, K. *et al.* Infectious Clones of Chikungunya Virus (La Réunion Isolate) for Vector Competence Studies. *Vector-Borne Zoonotic Dis.* **6**, 325–337 (2006).
58. Fischl, W. & Bartenschlager, R. High-Throughput Screening Using Dengue Virus Reporter Genomes. in *Methods in molecular biology (Clifton, N.J.)* vol. 1030 205–219 (2013).
59. Watt, A. *et al.* A Novel Life Cycle Modeling System for Ebola Virus Shows a Genome Length-Dependent Role of VP24 in Virus Infectivity. *J. Virol.* **88**, 10511–10524 (2014).
60. Sinzger, C. *et al.* Cloning and sequencing of a highly productive, endotheliotropic virus strain derived from human cytomegalovirus TB40/E. *J. Gen. Virol.* **89**, 359–368 (2008).
61. Hofmann, S. *et al.* Complex lipid metabolic remodeling is required for efficient hepatitis C virus replication. *Biochim. Biophys. Acta - Mol. Cell Biol. Lipids* **1863**, 1041–1056 (2018).
62. Papkalla, A., Münch, J., Otto, C. & Kirchhoff, F. Nef enhances human immunodeficiency virus type 1 infectivity and replication independently of viral coreceptor tropism. *J. Virol.* **76**, 8455–9 (2002).
63. Weil, T. *et al.* Advanced Molecular Tweezers with Lipid Anchors against SARS-CoV-2 and Other Respiratory Viruses. *JACS Au* **2**, 2187–2202 (2022).

Gating strategy

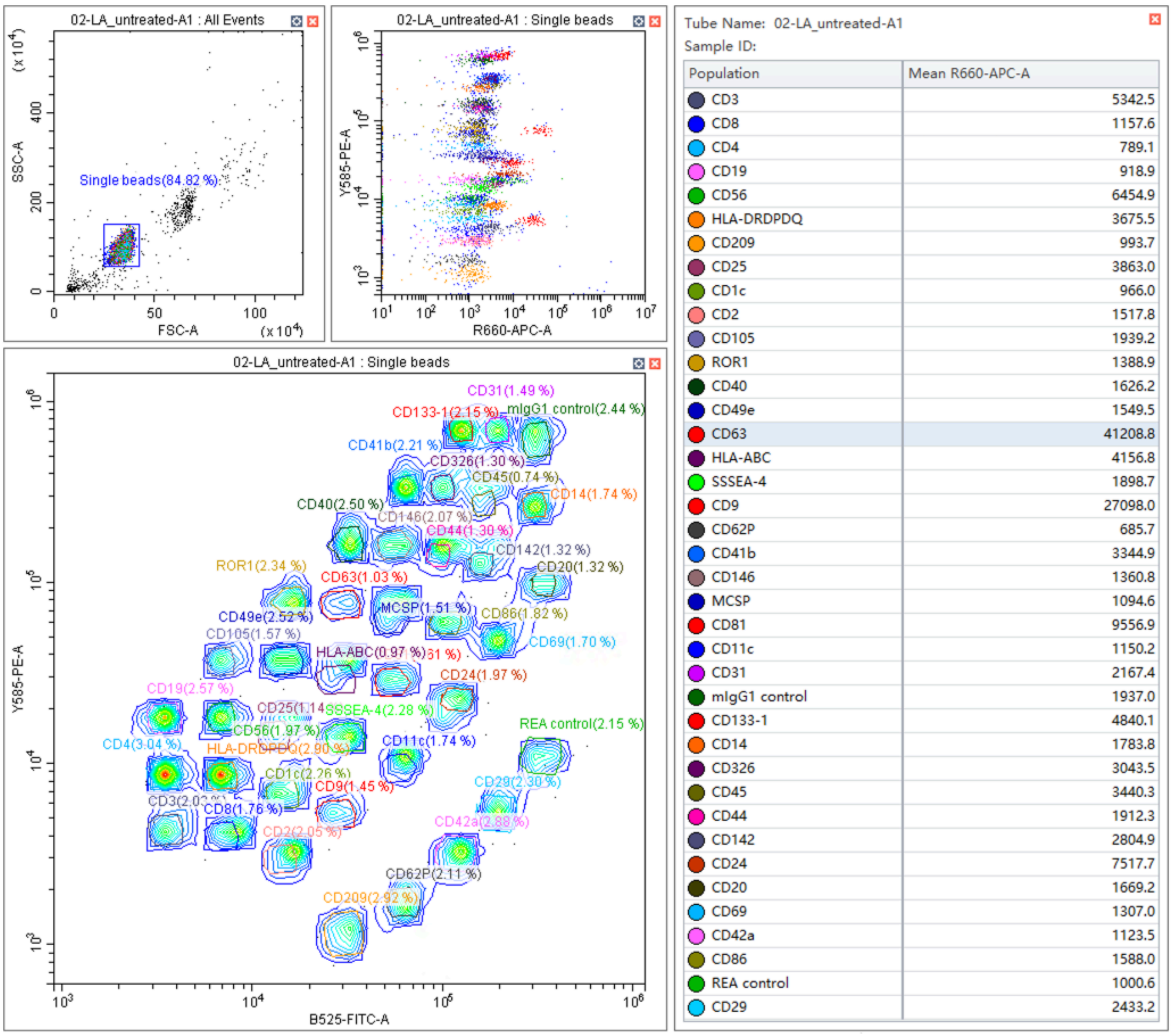

Tube Name: 02-LA_untreated-A1
Sample ID:

Population	Mean R660-APC-A
CD3	5342.5
CD8	1157.6
CD4	789.1
CD19	918.9
CD56	6454.9
HLA-DRDPDQ	3675.5
CD209	993.7
CD25	3863.0
CD1c	966.0
CD2	1517.8
CD105	1939.2
ROR1	1388.9
CD40	1626.2
CD49e	1549.5
CD63	41208.8
HLA-ABC	4156.8
SSSEA-4	1898.7
CD9	27098.0
CD62P	685.7
CD41b	3344.9
CD146	1360.8
MCSP	1094.6
CD81	9556.9
CD11c	1150.2
CD31	2167.4
mIgG1 control	1937.0
CD133-1	4840.1
CD14	1783.8
CD326	3043.5
CD45	3440.3
CD44	1912.3
CD142	2804.9
CD24	7517.7
CD20	1669.2
CD69	1307.0
CD42a	1123.5
CD86	1588.0
REA control	1000.6
CD29	2433.2

Gating strategy for bead-assisted flow cytometry as shown in Figure 3H, 4G, Extended Data Figure 4E. Representative raw data of EV-exposed PS using fluorescently tagged lactadherin. Single beads are first gated by SSC/FSC-A (top left) and individual beads containing antibodies directed against typical EV surface markers then separated by their endogenous PE/FITC-signal, enabling discrimination of 39 bead populations (37 markers + 2 isotype controls, large graph). The mean R660-APC/Alexa Fluro 647 fluorescence intensity of each bead population is then analyzed to reveal the proportion of PS-containing vesicles which simultaneously carry the protein marker of respective beads. Performed according to Wiklander et al. Systematic Methodological Evaluation of a Multiplex Bead-Based Flow Cytometry Assay for Detection of Extracellular Vesicle Surface Signatures. Front. Immunol. 9, 1326 (2018).

Uncut western blots

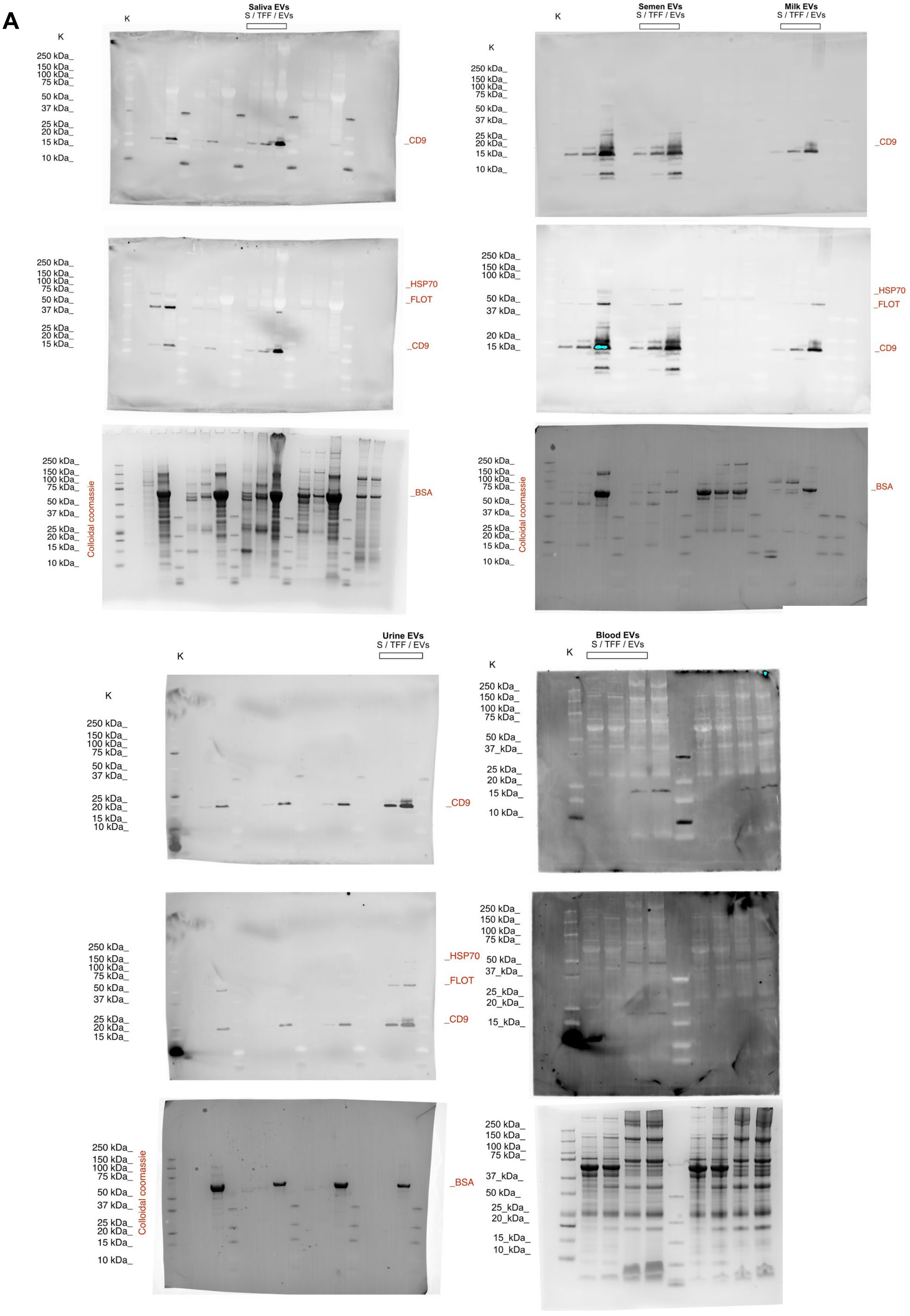

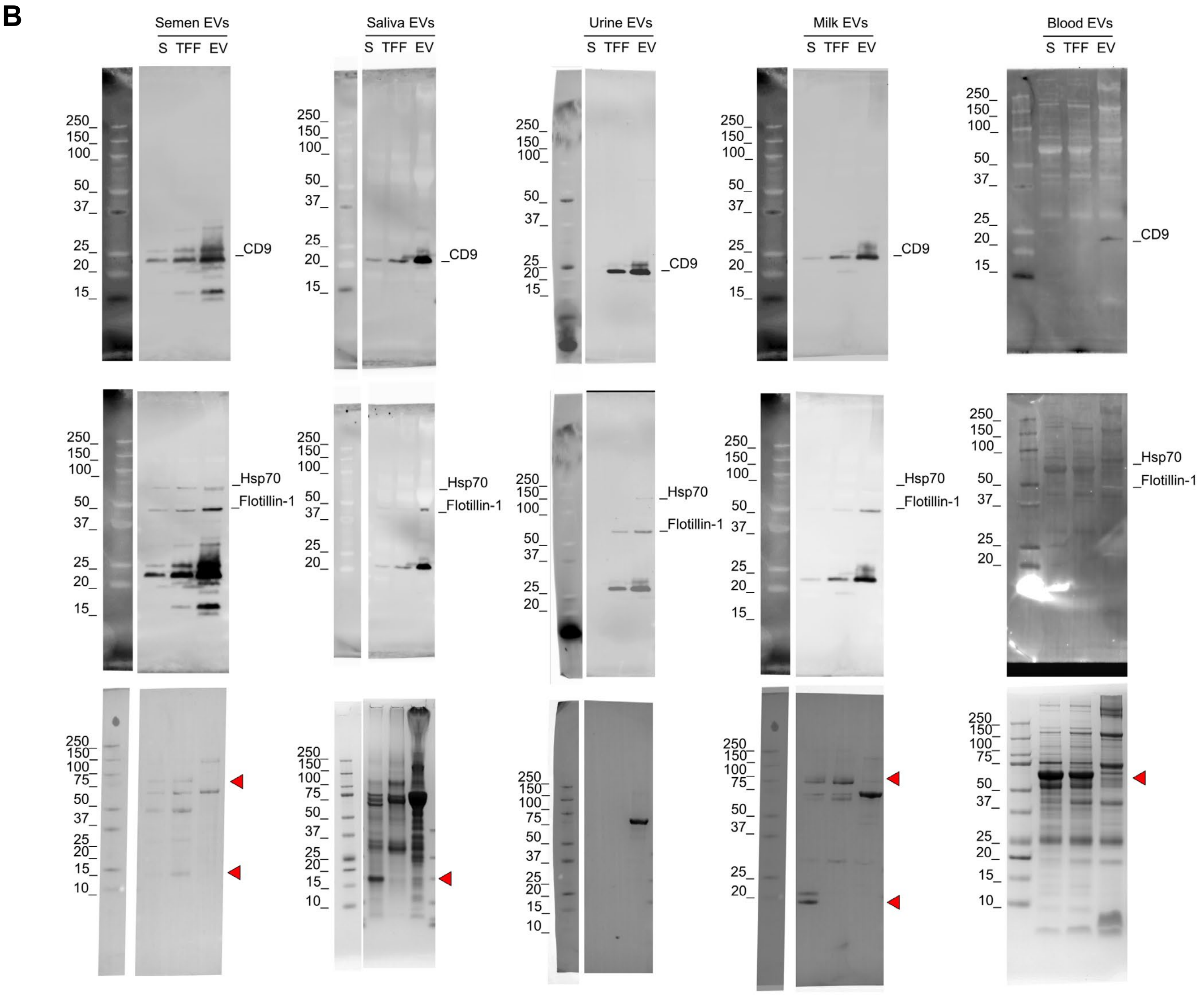

Uncut western blot membranes and total protein stain corresponding to cropped images shown in Figure 3B. Protein concentrations are 1 µg/lane (urine), 10 µg/lane (seminal plasma, breast milk, saliva, serum). For urine, the source fluid contained undetectable (< 25 µg/ml) protein levels and was thus used undiluted; TFF and BE-SEC fractions loaded are 1 µg/lane protein. For all preparations, membranes were stained for EV marker proteins CD9, Hsp70 and flotillin-1. Separate gels were stained for total protein by colloidal Coomassie (botton row). Note that after determining protein concentrations, all EV preparations were supplemented with BSA (0.1%, v/v) as a stabilizing agent (seen as a band at ~66 kDa in total protein stain). For serum, a sample without added BSA was separated on SDS-PAGE to visualize loss of HSA by purification. Proteins abundant in source fluids with notable reduction after TFF/BE-SEC are indicated by red triangles. Shown are A) uncut membranes and with B) and relevant lanes and markers.

Resource Table

REAGENT or RESOURCE	SOURCE	IDENTIFIER
**Antibodies**		
Anti-Flavivirus group antigen [D1-4G2-4-15 (4G2)]	Absolute Antibodies	Cat. # Ab00230-2.0 RRID:AB_2715504
CD9 (D8O1A) Rabbit mAb 13174	Cell Signaling Technology	Cat. # 13174 RRID: AB_2798139
Flotillin-1 (D2V7J) XP® Rabbit mAb 18634	Cell Signaling Technology	Cat. # 18634 RRID: AB_2773040
Goat anti-Mouse IgG (H+L) Secondary Antibody, HRP	Thermo Fisher Scientific	Cat. # 31430 RRID: AB_228307
Goat anti-mouse (H+L) Alexa Fluor 488	Thermo Fisher Scientific	Cat. # A11001 RRID: AB_2534069
Goat anti-Rabbit Affinity HRP	Thermo Fisher Scientific	Cat. # 31460 RRID: AB_228341
Goat anti-Mouse IgG (H+L) Superclonal Recombinant Secondary AB Alexa Fluor 647	Thermo Fisher Scientific	Cat. # A27029 RRID: AB_2536092
Goat anti-rabbit IgG StarBright 520	Bio-Rad	Cat. # 12005870 RRID: AB_2884949
Goat anti-rabbit IgG StarBright 700	Bio-Rad	Cat. # 12004162 RRID: AB_2721073
HSP70 (D69) Antibody 4876	Cell Signaling Technology	Cat. # 4876 RRID: AB_2119693
SARS-CoV/SARS-CoV-2 N 40143-MM05 Mouse mAb	SinoBiological	Cat. # 40143-MM05 RRID: AB_2827977
Axl Monoclonal Antibody (DS7HAXL), PE	Thermo Fisher Scientific	Cat. 12-1087-42 RRID: AB_2723961
CD9 (HI9a) FITC mouse mAb 312104	Biolegend	Cat. # 312104 RRID: AB_2075894
CD81 (TAPA-1) FITC mouse mAB 349504	Biolegend	Cat. # 349504 RRID: AB_2075894
CD63 (H5C6) FITC mouse mAB 353005	Biolegend	Cat. # 353005 RRID: AB_10898319
IgG1, κ (MOPC-21) mouse mAB 400109	Biolegend	Cat. # 400109 RRID: AB_2861401
IgG2a, κ (MOPC-173) mouse mAB 400207	Biolegend	Cat. # 400207 RRID: AB_2884007
**Chemicals, peptides, and recombinant proteins**		
18:1 (Δ9-Cis) PC (DOPC)	Avanti Polar Lipids	Cat. # 850375C
18:1 Cy5 PC	Avanti Polar Lipids	Cat. # 850483C
18:1 PS (DOPS)	Avanti Polar Lipids	Cat. # 840035C
Bovine Lactadherin Alexa 647	Haematologic Technologies, Inc.	Cat. # BLAC-ALEXA647
BSA	Gibco	Cat. # 15260-037
Choline	Sigma Aldrich	Cat. # C7527
Chloroform	Sigma Aldrich	Cat. # 34854

Clarity Max Western ECL Substrate	Bio-Rad	Cat. # 1705062
DSB-3	Bunz Lab	Choi et al., 2018[54]
DPBS	Gibco	Cat. # 14190-144
Dulbecco's Modified Eagle Medium (DMEM)	Gibco	Cat. # 41965039
Fetal bovine serum (FBS)	Gibco	Cat. # 10270106
GelCode Blue	Thermo Fisher Scientific	Cat. # 24590
HEPES	Gibco	Cat. # 15630-080
Hoechst 33342	Thermo Fisher Scientific	Cat. # H1399
Immobilon-FL PVDF	Merck	Cat. # IPFL00010
Isopropanol	Sigma Aldrich	Cat. # 33539
L-Glutamine	PAN-Biotech	Cat. # P04-80100
Methanol	Sigma Aldrich	Cat. # 32213
Methyl acetate	Sigma Aldrich	Cat. # 8.09711.100
Methyl-α-cyclodextrin	AraChem	Cat. # CDexA-076/BR
Non-essential amino acids	Gibco	Cat. # 11140-050
NuPAGE 4-12% BisTris gels	Thermo Fisher Scientific	Cat. # NP032
Paraformaldehyde 4%	VWR Chemicals	Cat. # 20910.294
Penicillin-Streptomycin	PAN-Biotech	Cat. # P06-07050
Phosphoserine	Selleckchem	Cat. # S5137
Phospolipase D from *Streptomyces c.*	Sigma Aldrich	Cat. # P0065
PS decarboxylase His6-Δ34PkPSD	Voelker lab	Choi et al., 2012[55]
Serine	Sigma Aldrich	Cat. # S4500
Sodium chloride	Sigma Aldrich	Cat. # 1.06404
Sodium hydroxide	Sigma Aldrich	Cat. # 415413
Sodium pyruvate	Gibco	Cat. # 11360-070
Sulfuric acid	Merck	Cat. # 1007311000
TMB substrate	SeraCare	Cat. # 5120-0078
TopFluor® PC	Avanti Polar Lipids	Cat. # 810281C
Tris(2-carboxyethyl)phosphine	Sigma Aldrich	Cat. # 646547
Triton X-100	Sigma Aldrich	Cat. # T9284
Trypsin-EDTA	PAN-Biotech	Cat. # P10-023100
Trypsin-EDTA	Gibco	Cat. # 25200-072
Tween-20	Sigma Aldrich	Cat. # P7949
**Critical commercial assays**		
CellTiter-Glo® Luminescent Cell Viability Assay	Promega	Cat. # G7571
MACSPlex Exosome Kit, human	Miltenyi Biotec	Cat. # 130-108-813
Pierce™ Rapid Gold BCA Protein Assay Kit	Thermo Fisher Scientific	Cat. # A53225
TaqMan™ Fast Virus 1-Step Master Mix	Thermo Fisher Scientific	Cat. # 4444436
LysoTracker™ Green DND-26	Thermo Fisher Scientific	Cat. # L7526

**Experimental models: Cell lines**		
BHK-21	ATCC	Cat. # CCL-10
Calu-3	ATCC	Cat. # HTB-55
ELVIS™	ATCC	Proffitt *et al.*, 1995[56]
HEK293T	ATCC	Cat# CRL3216
HFF	J. von Einem, Ulm University Medical Center	N/A
Huh-7	R. Bartenschlager, Heidelberg University	N/A
TZM-bl	NIBSC	Cat. # ARP5011
Vero E6	ATCC	Cat. # CRL-1586
**Experimental models: Organisms/strains**		
CHIKV	G. Simmons, UCSF	Tsetsarkin *et al.*, 2006[57]
DENV-R2a	N/A	Fischl et al., 2013[58]
EBOV-trVLP	Poehlmann Lab, GPC Göttingen	Watt *et al.*, 2014[59]
Ex vivo vaginal tissue	OB/GYN Clinic, Ulm University Medical Center	N/A
HCMV (TB40/E strain)	J. von Einem, Ulm University Medical Center	Sinzger *et al.*, 2008[60]
HCV (Jc1$^{p7\text{-}GLuc\text{-}2A\text{-}NS2}$)	N/A	Hofmann *et al.*, 2018[61]
HIV-1 92TH014	A. Papkalla, University of Erlangen	Papkalla *et al.*, 2002[62]
HSV-1 (F strain eGFP)	B. Kaufer, FU Berlin	N/A
HSV-2 (333 strain eGFP)	P.G. Spear, NU Chicago	N/A
SARS-CoV-2 B.1.617.2	H. Streeck, University of Cologne	Weil et al., 2023[63]
VSV	K.K. Conzelmann (Ludwig-Maximilians-University of Munich) and S. Whelan (Washington University St. Louis)	N/A
ZIKV (FB-GWUH-2016 strain)	O. Vapalahti, University of Helsiniki	N/A
ZIKV (MR766 strain)	J. Schmidt-Chanasit, Bernhard-Nocht-Institute Hamburg	N/A

**Oligonucleotides**		
ZIKV-F 5’-ACGGCYCTYGCTGGAGC-3’	Biomers.net	N/A
ZIKV-R 5’-GGAATATGACACRCCCTTCAAYCTAAG-3’	Biomers.net	N/A
ZIKV-P FAM-AGGCTGAGATGGATGGTGCAAAGGG-BNQ535	Biomers.net	N/A
**Software and algorithms**		
GraphPad Prism 9.5.1 and 10.0.2	GraphPad Software, Inc	www.graphpad.com
FIJI Version 2.3.0	N/A	www.imageJ.net/Fiji
CytExpert Version 2.3.0.84	Beckman Coulter, Inc	www.beckman.com
Huygens Professional 19.10	Scientific Volume Imaging	www.svi.nl
**Other**		
Extruder Set	Avanti Polar Lipids	Cat. # 610000
TFF hollow-fibre module 300 kDa MWCO	Repligen	Cat. # T06-E300-05-S
CaptoCore 700 BE-SEC	Cytiva	Cat. # 17548115
Vivaspin 20 100 kDa MWC0	Sartorius	Cat. # VS2042
Amicon Ultra-15 100 kDa MWCO	Sigma Aldrich	Cat. # UFC9110096
Amicon Ultra-0.5 10 kDa MWCO	Sigma Aldrich	Cat. # UFC501024
0.2 µm syringe filter	Merck Millipore	Cat. # SLGP033RB
Stericup 0.22 µm PVDF	Merck Millipore	Cat. # S2GVU05RE
HPTLC (High Performance TLC) Glass Plates	Sigma Aldrich	Cat. # Z740222
Hirschmann microcapillary pipette 1-5 µl	Sigma Aldrich	Cat. # Z611239
