# Supplementary material for: Phosphatidylserine-exposing extracellular vesicles in body fluids are an innate defence against apoptotic mimicry viral pathogens
Source: Nat Microbiol. 2024 Mar 25;9(4):905–21. doi: 10.1038/s41564-024-01637-6 (PMC10994849; doi:10.1038/s41564-024-01637-6)
Supplement: Supplementary file 2 — Reporting Summary [file 41564_2024_1637_MOESM2_ESM.pdf]

nature portfolio

Corresponding author(s): Prof. Dr. Janis Müller

Last updated by author(s): Jan 15, 2024

Reporting Summary

Nature Portfolio wishes to improve the reproducibility of the work that we publish. This form provides structure for consistency and transparency in reporting. For further information on Nature Portfolio policies, see our Editorial Policies and the Editorial Policy Checklist.

Statistics

For all statistical analyses, confirm that the following items are present in the figure legend, table legend, main text, or Methods section.

n/a	Confirmed	
☐	☒	The exact sample size (*n*) for each experimental group/condition, given as a discrete number and unit of measurement
☐	☒	A statement on whether measurements were taken from distinct samples or whether the same sample was measured repeatedly
☐	☒	The statistical test(s) used AND whether they are one- or two-sided<br>*Only common tests should be described solely by name; describe more complex techniques in the Methods section.*
☐	☒	A description of all covariates tested
☐	☒	A description of any assumptions or corrections, such as tests of normality and adjustment for multiple comparisons
☐	☒	A full description of the statistical parameters including central tendency (e.g. means) or other basic estimates (e.g. regression coefficient) AND variation (e.g. standard deviation) or associated estimates of uncertainty (e.g. confidence intervals)
☐	☒	For null hypothesis testing, the test statistic (e.g. *F*, *t*, *r*) with confidence intervals, effect sizes, degrees of freedom and *P* value noted<br>*Give P values as exact values whenever suitable.*
☒	☐	For Bayesian analysis, information on the choice of priors and Markov chain Monte Carlo settings
☐	☒	For hierarchical and complex designs, identification of the appropriate level for tests and full reporting of outcomes
☐	☒	Estimates of effect sizes (e.g. Cohen's *d*, Pearson's *r*), indicating how they were calculated

Our web collection on statistics for biologists contains articles on many of the points above.

Software and code

Policy information about availability of computer code

Data collection	Luminescence data (Cell-Titer Glo Luminescent viability assay, HIV-1 and HSV β-gal assay) was acquired using Simplicity 4.2 (Berthold Detection Systems). Absorbance-based assay data (viral protein immunodetection, BCA assay) was acquired using SoftMax Pro 7.0.3 (Molecular Devices). Flow cytometry data (Bead-assisted flow cytometry, HSV-1/2 infection assay) was acquired using CytExpert 2.3 (Beckman Coulter). Nanoparticle tracking data was acquired using ZetaVIEW using ZetaView Analyze 08.05.05 SP2 and 8.05.12 SP1. qPCR data (vaginal tissue block ZIKV replication) was acquired using StepOne Software 2.3. Confocal microscopy was performed using by confocal microscopy using a Zeiss LSM 710 and ZEN 2.3 (blue edition) software. DSB-3 reactions were recorded using a microplate fluorescence reader (BioTek Synergy) with Gen5 3.08.01. Nanoparticle flow cytometry was performed NanoAnalyzer (NanoFCM Co., Ltd, Nottingham, UK) and data collected using NanoFCM software (NF Profession V2.0). infected primary cells were imaged using Biotek Cytation with Gen5 Image+ 3.04.17. EVs preparation using ÄKTAstart chromatography system (Cytiva) was determined using A280 absorbance and UNICORN start 1.2.0.164.
Data analysis	Data was generally analyzed using Microsoft Excel for Mac, Version 16.71 and analyzed/visualized using GraphPad Prism for macOS Version 9.5.1. Statistical analyses were also performed using GraphPad Prism for macOS Version 9.5.1 and 10.0.2. For analysis of confocal microscopy data (attachment assay), Fiji (ImageJ) Version 2.3.0 was used with a macro that automatically identifies and counts local fluorescence maxima of a set of 1024 × 1024 px confocal images. Colocalization was evaluated using Huygens Professional software (Version 19.10) using Gaussian minimums for thresholding. FPLC chromatograms were analyzed using UNICORN start 1.2.0.164. (Cytiva). Flow cytometry data (Bead-assisted flow cytometry, HSV-1/2 infection assay) was analyzed using CytExpert 2.3 (Beckman Coulter). Nanoparticle tracking data was analyzed using ZetaVIEW Analyze 08.05.05 SP2 and 8.05.12 SP1. qPCR data (vaginal tissue block ZIKV replication) was analyzed using StepOne Software 2.3. NanoFCM data was evaluated by NanoFCM software (NF Profession V2.0). Illustrations created with BioRender.com.

For manuscripts utilizing custom algorithms or software that are central to the research but not yet described in published literature, software must be made available to editors and reviewers. We strongly encourage code deposition in a community repository (e.g. GitHub). See the Nature Portfolio guidelines for submitting code & software for further information.

Data

Policy information about availability of data

All manuscripts must include a data availability statement. This statement should provide the following information, where applicable:

- Accession codes, unique identifiers, or web links for publicly available datasets
- A description of any restrictions on data availability
- For clinical datasets or third party data, please ensure that the statement adheres to our policy

The data generated during this study are provided as Source Data and Supplementary Code. No original code was used. Any additional information required is available from the lead contact upon request.

Research involving human participants, their data, or biological material

Policy information about studies with human participants or human data. See also policy information about sex, gender (identity/presentation), and sexual orientation and race, ethnicity and racism.

Reporting on sex and gender	Extracellular vesicles used in this study were isolated from pooled body fluids of healthy human donors. For saliva and urine, donors included both male and female sex donors; gender data was not collected. Semen was obtained as excess material from volunteer donors at a fertility center, gender data was not determined. Breast milk was obtained from female sex nursing mothers, gender was not inquired. Blood (serum) was obtained from a commercial source, which was collected from male sex donors; gender was not inquired. Data was not analyzed for gender or sex-specific effects, as the described phenomenon is assumed to be sex-independent based on previous data (Conzelmann C, Groß R, Zou M, Krüger F, Görgens A, Gustafsson MO, El Andaloussi S, Münch J, Müller JA. Salivary extracellular vesicles inhibit Zika virus but not SARS-CoV-2 infection. J Extracell Vesicles. 2020 Aug 24;9(1):1808281. doi: 10.1080/20013078.2020.1808281. PMID: 32939236; PMCID: PMC7480612). Vaginal tissue used for ex vivo infection studies was obtained as excess material from patients undergoing surgical procedures for treatment of vaginal prolapse; gender was not inquired.
Reporting on race, ethnicity, or other socially relevant groupings	Extracellular vesicles used in this study were isolated from pooled body fluids of healthy human donors. Race, ethnicity, or other socially grouping was not inquired.<br>Vaginal tissue used for ex vivo infection studies was obtained as excess material from patients undergoing surgical procedures for treatment of vaginal prolapse; Race, ethnicity, or other socially grouping was not inquired.
Population characteristics	All samples used for extracellular vesicle isolation and vaginal tissue for infection were provided in an anonymized fashion and no data on age, genotypic information or past/current diagnosis or treatment of any disease was recorded.
Recruitment	Semen was obtained as excess material from volunteer donors at a fertility center, which may introduce a bias towards men with infertility. As the described phenonenon was donor independent in a previous study (Müller JA et al. Semen inhibits Zika virus infection of cells and tissues from the anogenital region. Nat Commun. 2018 Jun 7;9(1):2207. doi: 10.1038/s41467-018-04442-y. PMID: 29880824; PMCID: PMC5992203), we do not expect this to be be a confounding factor. Saliva and urine was collected from healthy, volunteering hospital employees. Breast milk was collected from volunteering nursing mothers. Participants were recruited via institution internal channels. There might be a bias towards german ethnicity, which we consider unlikely to impact the study outcome. Blood was obtained from a commercial source and no data on recruitment is available. Vaginal tissue was obtained as excess material from patients undergoing surgical procedures for treatment of vaginal prolapse with no selection bias other than the indication for surgery.
Ethics oversight	All experiments using human material were approved by the Ethics Comittee of Ulm University (Decision 88/17, 89/16, 89/17, 337/18, 131/16)

Note that full information on the approval of the study protocol must also be provided in the manuscript.

Field-specific reporting

Please select the one below that is the best fit for your research. If you are not sure, read the appropriate sections before making your selection.

☒ Life sciences ☐ Behavioural & social sciences ☐ Ecological, evolutionary & environmental sciences

For a reference copy of the document with all sections, see nature.com/documents/nr-reporting-summary-flat.pdf

Life sciences study design

All studies must disclose on these points even when the disclosure is negative.

Sample size	No statistical methods were used to calculate sample size for our biological experiments. Sample size was limited by availability of donor material (e.g. pooled body fluids necessary to yield sufficient concentrations of extracellular vesicles for downstream analysis).
Data exclusions	We did not exclude data from analysis
Replication	Experiments were performed with at least 3 biological replicates. Lipidomics analysis of EVs purified from pooled donors fluids was done with 2-3 technical replicates. Primary sample numbers dependent on the sample (n=2 for vaginal tissue; n=3-100 for most extracellular vesicle

sources, see Table S2). Where limited by availability of donor material, only one biological replicate was analyzed (breast milk). All replication attempts were successful.

Randomization	Randomization was not applicable as the study solely involved in vitro and ex vivo experiments, where controlled conditions negated the need for random assignment typical in clinical trials.
Blinding	Blinding was not performed due to the nature of the study involving only in vitro and ex vivo experiments, without clinical treatments.

Reporting for specific materials, systems and methods

We require information from authors about some types of materials, experimental systems and methods used in many studies. Here, indicate whether each material, system or method listed is relevant to your study. If you are not sure if a list item applies to your research, read the appropriate section before selecting a response.

Materials & experimental systems

n/a	Involved in the study	
☐	☒	Antibodies
☐	☒	Eukaryotic cell lines
☒	☐	Palaeontology and archaeology
☒	☐	Animals and other organisms
☒	☐	Clinical data
☒	☐	Dual use research of concern
☒	☐	Plants

Methods

n/a	Involved in the study	
☒	☐	ChIP-seq
☐	☒	Flow cytometry
☒	☐	MRI-based neuroimaging

Antibodies

Antibodies used

Anti-Flavivirus group antigen [D1-4G2-4-15 (4G2)] Absolute Antibody Cat. # Ab00230-2.0
RRID:AB_2715504 (1:5,000 for WNV in-cell ELISA; 1;10,000 for ZIKV in-cell ELISA and confocal microscopy)

CD9 (D8O1A) Rabbit mAb 13174 Cell Signaling Technology Cat. # 13174
RRID: AB_2798139 (1:1,000 in western blotting)

Flotillin-1 (D2V7J) XP® Rabbit mAb 18634 Cell Signaling Technology Cat. # 18634
RRID: AB_2773040 (1:1,000 in western blotting)

Goat anti-Mouse IgG (H+L) Secondary Antibody, HRP Thermo Fisher Scientific Cat. # 31430
RRID: AB_228307 (1:10,000 for ZIKV and WNV in-cell ELISA; 1:15,000 for SARS-CoV-2 in-cell ELISA)

Goat anti-mouse (H+L) Alexa Fluor 488 Thermo Fisher Scientific Cat. # A-11001
RRID: AB_2534069 (1:400 in ZIKV immunoimaging)

Goat anti-Rabbit Affinity HRP Thermo Fisher Scientific Cat. # 31460
RRID: AB_228341 (1:10,000 in western blotting)

Goat anti-mouse IgG (H+L) Superclonal Recombinant Secondary AB Alexa Fluor 647 Thermo Fisher Scientific Cat. # A27029
RRID: AB_2536092 (1:1,000 in ZIKV attachment imaging)

Goat anti-rabbit IgG StarBright 520 Bio-Rad Cat. # 12005870
RRID: AB_2884949 (1:2,500 in western blotting)

Goat anti-rabbit IgG StarBright 700 Bio-Rad Cat. # 12004162
RRID: AB_2721073 (1:2,500 in western blotting)

HSP70 (D69) Antibody 4876 Cell Signaling Technology Cat. # 4876
RRID: AB_2119693 (1:1,000 in western blotting)

SARS-CoV/SARS-CoV-2 N 40143-MM05 Mouse mAb SinoBiological Cat. # 40143-MM05
RRID: AB_2827977 (1:5,000 in SARS-CoV-2 in-cell ELISA)

Axl Monoclonal Antibody (DS7HAXL), PE Thermo Fisher Scientific Cat. 12-1087-42
RRID: AB_2723961 (120 ng/ml in confocal microscopy)

CD9 (HI9a) FITC mouse mAb 312104, Biolegend, Cat. # 312104
RRID: AB_2075894 (12.5 µM in nanoflow cytometry)

CD81 (TAPA-1) FITC mouse mAB 349504, Biolegend, Cat. # 349504
RRID: AB_2075894 (12.5 µM in nanoflow cytometry)

CD63 (H5C6) FITC mouse mAB 353005, Biolegend, Cat. # 353005
RRID: AB_10898319 (12.5 µM in nanoflow cytometry)

IgG1, κ (MOPC-21) mouse mAB 400109, Biolegend, Cat. # 400109
RRID: AB_2861401 (12.5 µM in nanoflow cytometry)

IgG2a, κ (MOPC-173) mouse mAB 400207, Biolegend, Cat. # 400207
RRID: AB_2884007 (12.5 µM in nanoflow cytometry)

Validation

Antibodies were validated by the manufacturers.

Eukaryotic cell lines

Policy information about cell lines and Sex and Gender in Research

Cell line source(s)

BHK-21 - obtained from ATCC Cat. # CCL-10
Calu-3 - obtained from M. Frick, Ulm University (ATCC # HTB-55)
HEK293T - obtained from ATCC Cat. # CRL3216
HFF - primary, obtained from J. von Einem, Ulm University Medical Center
TZM-bl (HeLa derived) - obtained from NIBSC Cat. # ARP5011
Vero E6 - obtained from ATCC Cat. # CRL-1586
Huh-7 - obtained from R. Bartenschlager, Heidelberg University, described in Windisch et al., 2005 and Lohmann and Bartenschlager 2014
ELVIS - obtained from ATCC described in Profitt et al, 1995

Authentication

Cells were purchased from indicated companies or labs and used without further authentication.

Mycoplasma contamination

All cells were regularly tested for mycoplasma contamination and tested negative.

Commonly misidentified lines
(See ICLAC register)

No commonly misidentified cell lines were used.

Plants

Seed stocks

Report on the source of all seed stocks or other plant material used. If applicable, state the seed stock centre and catalogue number. If plant specimens were collected from the field, describe the collection location, date and sampling procedures.

Novel plant genotypes

Describe the methods by which all novel plant genotypes were produced. This includes those generated by transgenic approaches, gene editing, chemical/radiation-based mutagenesis and hybridization. For transgenic lines, describe the transformation method, the number of independent lines analyzed and the generation upon which experiments were performed. For gene-edited lines, describe the editor used, the endogenous sequence targeted for editing, the targeting guide RNA sequence (if applicable) and how the editor was applied.

Authentication

Describe any authentication procedures for each seed stock used or novel genotype generated. Describe any experiments used to assess the effect of a mutation and, where applicable, how potential secondary effects (e.g. second site T-DNA insertions, mosiacism, off-target gene editing) were examined.

Flow Cytometry

Plots

Confirm that:

☒ The axis labels state the marker and fluorochrome used (e.g. CD4-FITC).

☒ The axis scales are clearly visible. Include numbers along axes only for bottom left plot of group (a 'group' is an analysis of identical markers).

☒ All plots are contour plots with outliers or pseudocolor plots.

☒ A numerical value for number of cells or percentage (with statistics) is provided.

Methodology

Sample preparation

For flow cytometric determination of HSV-1 and -2 infection by virus-encoded eGFP, cells (Vero E6) were trypsinized (0.25% Trypsin), transferred to V-well plates, centrifuged and fixed in 4% PFA (in PBS) for 1h at 4°C. Fixed cells were then acquired by flow cytometry.

For determination of EV surface antigens using bead-assisted flow cytometry, the MACSPlex Exosome Kit, human (Milteny Biotec) was used as described by the manufacturer and bead-bound EVs stained with the included anti-Tetraspanin cocktail or Alexa 647-conjugated bovine Lactaderine (BLAC-A647, Haematologic Technologies).

For nano-flow cytometry, 12.5 µM of corresponding antibodies in 50 µl TE or 5 µg/ml lactadherin-A647 was used. After removing antibody aggregates by centrifugation at 12,000×g for 10 min, the supernatant was added to 2 × 10^8 particles, followed by incubation for 12 h at 37∘C under constant shaking and washing with 1 ml 1x TE buffer by ultracentrifugation at 110,000×g for 45 min at 4∘C (Beckman Coulter MAX-XP centrifuge, TLA-45 rotor; Beckman Coulter, Krefeld, Germany). The pellet was resuspended in 50 µl 1x TE buffer for nFCM analysis. Monodisperse silica beads (NanoFCM Co., Ltd, Nottingham, UK) of four different diameters (68 nm; 91 nm; 113 nm; 155 nm) served as size reference standards. Freshly filtered (0.1 µm) 1x TE buffer pH 7.4 was analyzed to define the background signal, which was subtracted from all other measurements. The EV samples were diluted with filtered (0.1 µm) 1x TE buffer, resulting in a particle count in the optimal range of 2,500–12,000 events.

Instrument

Cytoflex LX, Beckman Coulter; Flow NanoAnalyzer , NanoFCM

Software

CytExpert 2.3; NF Profession V2.0

Cell population abundance

For analysis of cell lines and beads purity is not applicable. Extracellular vesicles preparation is thorougly described in the manuscript.

Gating strategy

Single cells and beads were gated based on FSC/SSC plots. The gating strategy for MACSplex beads was previously shown (Wiklander OPB, Bostancioglu RB, Welsh JA, Zickler AM, Murke F, Corso G, Felldin U, Hagey DW, Evertsson B, Liang XM, Gustafsson MO, Mohammad DK, Wiek C, Hanenberg H, Bremer M, Gupta D, Björnstedt M, Giebel B, Nordin JZ, Jones JC, El Andaloussi S, Görgens A. Systematic Methodological Evaluation of a Multiplex Bead-Based Flow Cytometry Assay for Detection of Extracellular Vesicle Surface Signatures. Front Immunol. 2018 Jun 13;9:1326. doi: 10.3389/fimmu.2018.01326. PMID: 29951064; PMCID: PMC6008374.) No gating was performed for nano-flow cytometry, all events are shown.

☒ Tick this box to confirm that a figure exemplifying the gating strategy is provided in the Supplementary Information.
